# Impact of database choice and confidence score on the performance of taxonomic classification using Kraken2

**DOI:** 10.1007/s42994-024-00178-0

**Published:** 2024-07-31

**Authors:** Yunlong Liu, Morteza H. Ghaffari, Tao Ma, Yan Tu

**Affiliations:** 1 Key Laboratory of Feed Biotechnology of the Ministry of Agricultural and Rural Affairs, Institute of Feed Research, Chinese Academy of Agricultural Sciences, Beijing, 100081 China; 2 Institute of Animal Science, Physiology Unit, University of Bonn, Bonn, 53115 Germany

**Keywords:** Metagenome, Taxonomic classification, Kraken2, Reference database, Confidence score

## Abstract

Accurate taxonomic classification is essential to understanding microbial diversity and function through metagenomic sequencing. However, this task is complicated by the vast variety of microbial genomes and the computational limitations of bioinformatics tools. The aim of this study was to evaluate the impact of reference database selection and confidence score (CS) settings on the performance of Kraken2, a widely used k-mer-based metagenomic classifier. In this study, we generated simulated metagenomic datasets to systematically evaluate how the choice of reference databases, from the compact Minikraken v1 to the expansive nt- and GTDB r202, and different CS (from 0 to 1.0) affect the key performance metrics of Kraken2. These metrics include classification rate, precision, recall, F1 score, and accuracy of true versus calculated bacterial abundance estimation. Our results show that higher CS, which increases the rigor of taxonomic classification by requiring greater k-mer agreement, generally decreases the classification rate. This effect is particularly pronounced for smaller databases such as Minikraken and Standard-16, where no reads could be classified when the CS was above 0.4. In contrast, for larger databases such as Standard, nt and GTDB r202, precision and F1 scores improved significantly with increasing CS, highlighting their robustness to stringent conditions. Recovery rates were mostly stable, indicating consistent detection of species under different CS settings. Crucially, the results show that a comprehensive reference database combined with a moderate CS (0.2 or 0.4) significantly improves classification accuracy and sensitivity. This finding underscores the need for careful selection of database and CS parameters tailored to specific scientific questions and available computational resources to optimize the results of metagenomic analyses.

**Supplementary Information:**

The online version contains supplementary material available at 10.1007/s42994-024-00178-0.

## Introduction

Metagenomic sequencing techniques provide a comprehensive understanding of the diversity and functional potential of microbial communities (Lu et al. 2022). However, achieving precise identification of microbial composition is challenging as it requires genomic data from a mixed species community rather than a pure species isolate (Quince et al. 2017). Taxonomic classification is a crucial step in identifying the membership of microbial communities in a sample, and several bioinformatic tools have been developed to address this need. These include alignment-based methods such as BLAST (Johnson et al. 2008; Camacho et al. 2009), k-mer-based approaches such as Kraken2 (Wood et al. 2019) and large-scale machine learning-based methods (Mathieu et al. 2022).

While BLAST is known for its sensitivity in metagenomic alignment, it is computationally intensive and impractical for processing the millions of reads typically generated in metagenomic studies (Wood and Salzberg 2014; Zielezinski et al. 2017). Kraken2, on the other hand, examines K-mers within a query sequence and consults a reference database by mapping these K-mers to the lowest common ancestor (LCA) of all genomes containing a given K-mer (Wood et al. 2019). This method provides a good balance between speed and accuracy and is, therefore, suitable for large-scale metagenomic analyses.

The choice of reference database is a crucial factor in the taxonomic classifications of Kraken2 (Smith et al. 2022). Ideally, the reference database should include a wide range of microbial genomes to ensure broad coverage of potential organisms in a sample. A popular choice is the comprehensive NCBI RefSeq Complete Genomes and the nt database for high-quality nucleotides (NCBI Resource Coordinators 2014; O’Leary et al. 2016; Méric et al. 2019). However, the size of these databases can pose significant computational challenges, as the storage requirements can exceed 100 GB (Ye et al. 2019). Smaller databases such as Minikraken or the use of the ‘–max-db-size’ parameter to limit the database size offer alternatives but can come at the expense of scalability.

A special feature of Kraken2 is the user-defined confidence score (CS) within the interval [0–1], which controls the taxonomic labeling process. If a label’s score, which is determined by the proportion of matching k-mers, reaches or exceeds the threshold value, it is included in the taxonomic tree. However, if the score does not reach the threshold, the reads remain unclassified. A higher CS value indicates a higher stringency, i.e. a larger proportion of k-mers must match for taxonomic classification. This approach reduces the probability of misclassification but can lead to a higher number of unclassified reads if no consensus is reached. Most metagenomic studies using Kraken2 for taxonomic analysis set the CS to 0.2 or 0.4 (Loomis et al. 2021; Li et al. 2022a; Collins et al. 2023; Pereira-Marques et al. 2024). However, some studies use a higher CS of 0.8 (Yan et al. 2022) or even 1.0 for the identification of certain pathogens (Doster et al. 2019). Higher CS settings improve precision but can reduce sensitivity as a greater proportion of k-mers must match for a read to be classified, resulting in more reads remaining unclassified.

Previous studies have shown different effects of CS on classification performance. For example, using a higher CS can significantly increase precision but result in lower sensitivity as more reads remain unclassified (Wright et al. 2023). Despite these findings, there is no definitive guidance for selecting the optimal CS, leaving this decision to the researcher preference. This lack of standardized guidelines often leads to overlooking the impact on the accuracy of taxonomic classification.

In the present study, we systematically investigated the impact of the choice of reference database and CS on classification rate, precision, recall, F1 score and the difference between the ‘true’ and ‘estimated’ relative abundance using simulated datasets with known bacterial composition. Our aim was to evaluate how these factors influence the overall classification performance of Kraken2 and gain insights into optimizing its use for accurate taxonomic profiling. This study fills the knowledge gap regarding the influence of CS and database choice on metagenomic analysis results and provides guidance for researchers to select appropriate parameters for their specific needs.

## Results

### Impact on classification rate

**Fig. 1:**
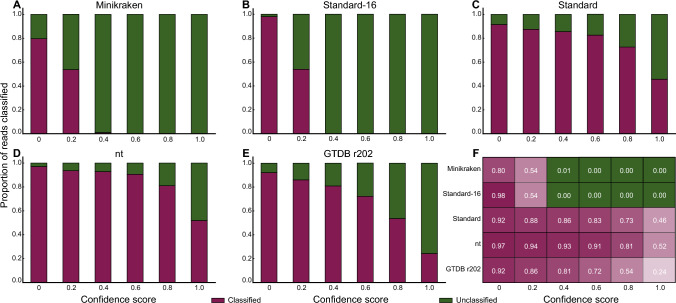
The proportion of reads classified with different reference databases (**A** Minikraken; **B** Standard-16; **C** Standard; **D** nt; **E** GTDB r202) and confidence scores (0, 0.2, 0.4, 0.6, 0.8, and 1.0) in Kraken2 using simulated metagenomic datasets. The results from the k2report provided us with percentages of classified and unclassified reads. The classification level referred to in our analysis is the “Root” level, the highest biological classification level. Purple represents classified data, while green represents unclassified data. In Fig. 1F, all data represent the proportion of classified reads, with varying degrees of purple transparency representing differences in classified proportions. In both Minikraken and Standard-16 databases, the proportion of classified reads approach zero when the confidence score exceeds 0.4, thus green color is used to distinguish

When using the Minikraken and Standard-16 databases, the classification rate decreased sharply with increasing CS, and no reads could be classified at a CS above 0.4 (Fig. 1A and B; Supplementary Table 1). In comparison, a considerable number of sequences could be classified when the CS was 1.0 and the nt, Standard and GTDB r202 databases were used (Fig. 1C–E; Supplementary Table 1). At a CS of 0, the classification rate was lowest when the Minikraken database was used (Fig. 1F; Supplementary Table 1). At a CS of 0.2, 0.4 or 0.6, the classification rate was higher with the Standard, nt and GTDB r202 databases than with the other two databases (Fig. 1F). At a CS of 0.8 or 1.0, the classification rate was higher with the nt databases than with the other databases except the standard database (Fig. 1F).

### Impact on precision, recall, and F1 score

**Fig. 2:**
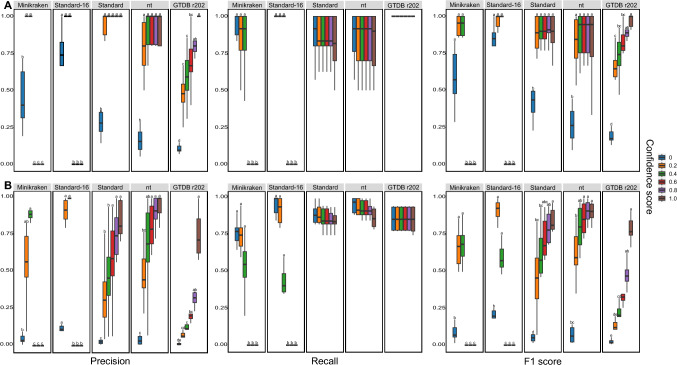
Precision, Recall, and F1 score of classification with different databases (Minikraken, Standard-16, Standard, nt, and GTDB r202) and confidence scores (0, 0.2, 0.4, 0.6, 0.8, and 1.0) at phylum (**A**) and species (**B**) level. Different letters indicate statistically significant differences (*P* ≤ 0.05). See Supplementary Table 2–4 for raw values and significance analysis

At both phylum and species levels (Fig. 2; Supplementary Table 2), the precision of classification significantly with increasing CS for the Standard, nt and GTDB r202 databases, but decreased to 0 for the Minikraken and Standard-16 databases when CS was 0.6 or higher. When CS was 0, the precision of classification was higher for the Standard-16 database at phylum and species levels. The GTDB r202 database provided lower precision of classification when CS was 0.2 or 0.4 at the phylum and species levels. When CS was 0.6 or 0.8, classification precision did not differ between the Standard, nt and GTDB r202 databases at the phylum level, but was lower for the GTDB r202 database than for the nt database at the species level. No difference was observed in the precision of classification when CS was 1.0 and Standard, nt or GTDB r202 databases were used at both phylum and species level.

At both stem and species levels (Fig. 2; Supplementary Table 3), the recall of classification was not affected by CS when the Standard, nt or GTDB r202 databases were used, or when the Minikraken and Standard-16 databases were used when CS was 0, 0.2 and 0.4, respectively. However, for the Minikraken and Standard-16 databases, the recall of classification decreased to 0 when CS was above 0.4. At the phylum level, there was no significant difference in classification recall when using different databases when CS was 0, 0.2 or 0.4, and when using the Standard, nt or GTDB r202 databases when CS was 0.6, 0.8 or 1.0. At the species level, the recall of classification was higher when classifying with the Standard-16 and nt databases than with the Minikraken database when CS was 0 or 0.2, and higher with the Standard, nt or GTDB r202 databases when CS was 0.4 or higher.

Similar to the precision of classification, F1 score increased with increasing CS when using Standard, nt or GTDB r202 databases, but decreased to 0 when CS was 0.6 or higher at both phylum and species levels for the Minikraken and Standard-16 databases (Fig. 2; Supplementary Table 4). When CS was 0, the F1 score was higher using the Standard-16 databases at phylum and species levels. The GTDB r202 database yielded a lower F1 score when CS was 0.2 or 0.4 at the phylum and species levels. When CS was 0.6 or 0.8, the F1 score did not differ when using Standard, nt, or GTDB r202 databases at the phylum level, but was lower when using the GTDB r202 database than when using the nt database at the species level. No difference was observed in F1 score when CS was 1.0 and Standard, nt or GTDB r202 databases were used at both phylum and species level.

### Impact on the difference between calculated and true relative abundance of bacterial

**Fig. 3:**
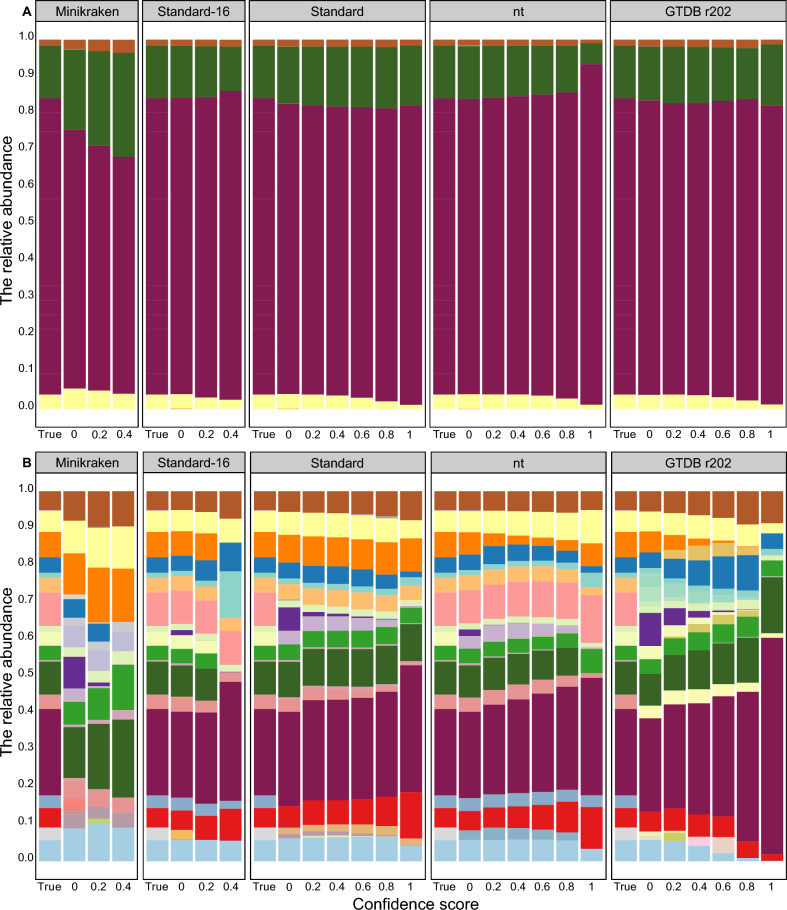
The bacterial composition and relative abundance classified under different databases (Minikraken, Standard-16, Standard, nt, and GTDB r202) and confidence scores (0, 0.2, 0.4, 0.6, 0.8, and 1.0) compared with true datasets at phylum (**A**) and species (**B**) level. ‘True’ represents the simulated bacterial composition and relative abundance. After classification using Kraken2, the results display different compositions and proportions across various databases and confidence scores. At the species level, for each confidence score, we retained only the top 20 species and classified the remaining species as ‘others’ (shown in purple in Fig. 3B)

**Fig. 4:**
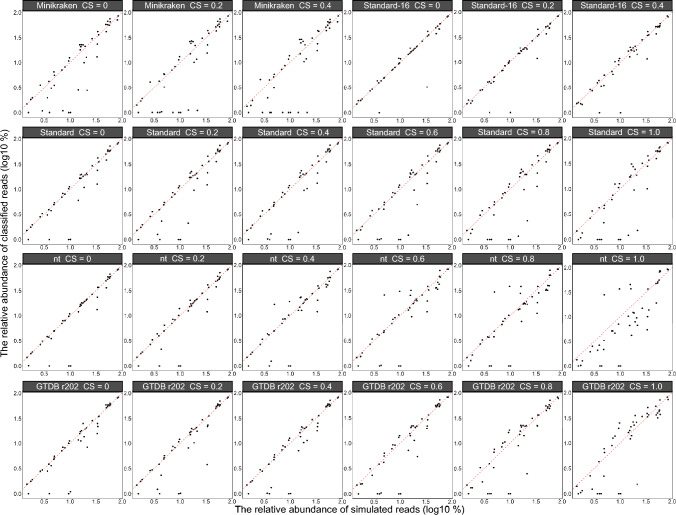
The difference between the relative abundance (log10 transformed) of bacterial phyla calculated and the true relative abundance (log10 transformed) of bacterial phyla at different databases (Minikraken, Standard-16, Standard, nt, and GTDB r202) and confidence scores (0, 0.2, 0.4, 0.6, 0.8, and 1.0). The red dotted line indicates that the difference is 0. The closer the black points are to the red dotted line, the closer the relative abundance of bacteria after classification is to the true relative abundance

**Fig. 5:**
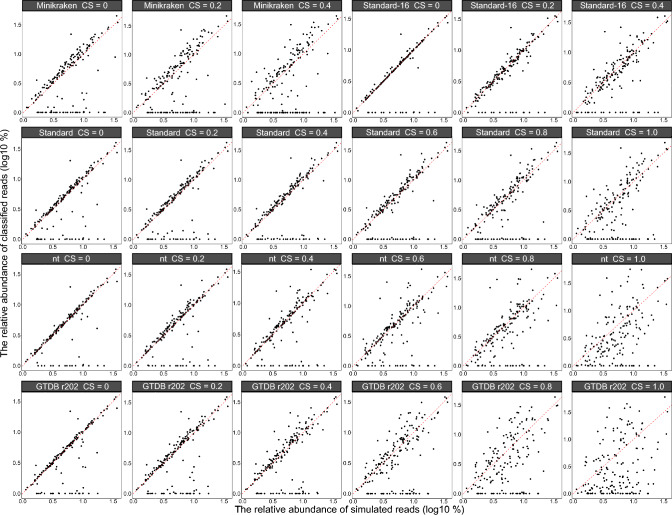
The difference between the relative abundance (log10 transformed) of bacterial species calculated and the true relative abundance (log10 transformed) of bacterial species at different databases (Minikraken, Standard-16, Standard, nt, and GTDB r202) and confidence scores (0, 0.2, 0.4, 0.6, 0.8, and 1.0). The red dotted line indicates that the difference is 0. The closer the black points are to the red dotted line, the closer the relative abundance of bacteria after classification is to the true relative abundance

After classifying true datasets with kraken2 with different databases and CS, the composition and relative abundance of the classified bacteria show variations (Fig. 3). In each database and CS category, we kept the top 20 species and classified the remaining species as ‘other’’ (black section, Fig. 3B). Variations in species composition and relative abundance were observed with increasing CS. At both phylum (Fig. 4; Supplementary Table 5) and species levels (Fig. 5; Supplementary Table 5), the difference between the calculated and true relative abundance of bacteria increased significantly with increasing CS, except when using the Standard database at the phylum level. The difference was significantly higher when using the Minikraken database at any CS and when using the Standard 16 database when CS was 0.6 or higher at both phylum and species levels.

## Discussion

Metagenomic sequencing techniques have been extensively applied in medicine, agriculture and environmental sciences (Riesenfeld et al. 2004; Chiu and Miller 2019; Li et al. 2022a, b) and provide crucial insights into the composition and functionality of microbial communities. Taxonomic classification is a crucial step in metagenomic analysis (Ames et al. 2013), and Kraken2 has become a widely used tool for aligning short reads to a microbial reference database (Ye et al. 2019). Kraken2 aligns each k-mer in a sequencing read to a reference database, which stores the taxonomy ID for each k-mer and then assigns a taxonomic designation based on the most accurate match (Breitwieser et al. 2018). The CS determines the proportion of k-mers in a read that must have the same taxonomic assignment, which is typically determined by the lowest common ancestor (LCA) algorithm in the taxonomic tree (Wood et al. 2019).

Several benchmarking studies have evaluated the performance of Kraken2 and other classifiers (Ye et al. 2019; Cárdenas et al. 2022; Govender and Eyre 2022; Jurado-Rueda et al. 2023). However, there is a lack of studies investigating how specific parameters of a classifier, such as the CS in Kraken2, together with the choice of reference database, influence the overall performance of taxonomic classification. A recent study by Wright et al. (2023) investigated the effect of CS on classification performance (e.g. precision, recall and F1 score) but did not consider the choice of reference database or how CS together with the reference databases affected the difference between true and post-classified relative abundance, as human-associated and environmental datasets were used rather than simulated datasets.

Our study found that the choice of reference database can lead to significant variability in the proportion of classified reads in the simulated datasets. This variability can be attributed to the microbial composition of the database itself. Larger databases with broader microbial genome coverage tend to have a more comprehensive species representation (Rodriguez-R and Konstantinidis 2014; Breitwieser et al. 2019). Compared to the choice of reference database, CS has a greater impact on classification rates. A higher CS requires a stronger agreement between the k-mers within a read for taxonomic classification, resulting in fewer reads meeting the higher threshold. This strict standard naturally reduces the classification rate as fewer reads meet the higher CS. For example, the decrease in classification rate when using the Standard and nt databases compared to the GTDB r202 database was less significant when the CS was increased; at CS = 1.0, only 24% of reads were classified using the GTDB r202 database. In addition, the classification rate for the Minikraken and Standard-16 databases dropped to almost zero when CS was above 0.4. This could be due to the lower number of k-mers in these databases. For example, the Minikraken database contains only 5% of the k-mers from the original Standard databases, which reduces the number of reads successfully mapped to the taxonomy tree.

In addition to the classification rate, our results also indicated that the choice of reference database and CS influences the precision, recall and F1 score of the classification. Due to the comprehensive genomic information within larger databases, particularly including massive closely related species, setting a CS to 0 could result in lower precision and F1 score, especially at the species level. On the other hand, a slight increase in CS had the potential to significantly increase precision and F1 score in our study. For example, the classification precision and F1 score increased from 0.16 to 0.76 and from 0.07 to 0.58, respectively, when the CS was increased from 0 to 0.2 using the default database at the species level. However, it should be noted that further increasing the CS from 0.2 did not improve precision and could even result in precision and F1-score that were not different from zero when a higher CS was chosen (e.g. 0.6 in this study) for databases of a smaller size such as the Minikraken and Standard 16 databases, suggesting that a balance between classification rate as well as precision/F1-score should be established. On the other hand, recall is an important metric for evaluating the sensitivity of metagenomic data classification. It provides an objective assessment of how well a classifier can identify microbial species in a sample. A higher recall value indicates that a significant proportion of species are correctly identified. Our results show that recall remained consistent across all phylum and species levels when the larger reference databases (Standard, nt and GTDB r202) were used independently of CS. Similarly, Wright et al. (2023) showed that recall did not vary greatly when using the NCBI RefSeq Complete V205 database in the range of 0 to 1.0, suggesting that recall may be less influenced by CS compared to precision and F1 score.

The relative abundance of a taxon is particularly important in metagenomic sequencing studies. Our results show that the difference between the calculated and the true relative abundance of bacteria increases with increasing CS regardless of the chosen database. This could be due to the fact that at higher CS, numerous reads are filtered out, leading to a significant increase in unclassified reads and consequently fluctuations in relative abundance. We also found that this difference was more significant when using the Minikraken database than when using other databases with increasing CS at the phylum level. The Standard 16 database, although a smaller database, yielded a similar difference when CS was 0.2 compared to the difference found with larger databases at the phylum and species level. For larger size databases, although the difference also increased with increasing CS, the Standard database was found to be more robust compared to the other two databases and showed a smaller variation (from 3.03 to 4.90% at the phylum level and from 1.73 to 3.21% at the species level). It should be noted that as CS increases, a number of classified reads may naturally be filtered out, resulting in a higher difference between the calculated and true relative abundance. However, the proportion of these reads could be either relatively small (< 0.0001%) or biologically irrelevant. With increasing CS, the number of false-positive species is likely to decrease significantly. However, the number of unclassified reads may increase, leading to a loss of true positive species. In addition, when using large databases, the inclusion of more species can lead to a higher number of false positives, particularly those that are closely related to the true species. In this case, a small increase in CS can effectively eliminate the number of false-positive reads without having a major impact on the reliability of the microbial composition of a dataset.

Most of the previous studies used default CS (0) of Kraken2, with some recent studies applying a more stringent CS such as 0.05 (Ring et al. 2023) or 0.1 (Szóstak et al. 2022; Rumore et al. 2023) using human-associated or environmental samples. Accurate identification of pathogens is essential in studies investigating the interaction of microbes with hosts. Researchers often use a CS of 0.8 or higher, even when using the maximum value, to classify species and improve annotation accuracy (Doster et al. 2019; Yan et al. 2022). It is also worth noting that some powerful pipelines usually include multiple tools to achieve different functions such as taxonomic classification or functional analysis. For example, AMR +  + , a bioinformatics pipeline for identifying antimicrobial resistance genes from metagenomics sequence data (Bonin et al. 2023), includes Kraken2 as a classifier for microbial composition analysis. In contrast to the default CS (0) used in most studies, the pipeline assumes the highest CS value (1.0) by default. While this can minimize the false positive rate, some ‘genuine’ taxa may be excluded. If you are using such pipelines, you should review the default settings of each tool enwrapped in the pipeline and make appropriate adjustments depending on the specific aim of your studies. There are no precise guidelines for the choice of CS for Kraken2 and it is mainly determined by the researcher's personal choice or the default value. Therefore, more consideration should be given to the choice of CS and its impact on the research results.

Our results suggest that the use of a more comprehensive reference database (e.g. Standard or nt) in combination with a modest CS of 0.2 or 0.4 can significantly improve the accuracy and sensitivity of classification. However, since our study was based on simulated datasets, these results need to be further validated with real datasets with larger sample sizes. In addition, limited access to powerful computing resources may be a significant obstacle for some researchers, preventing them from effectively utilizing large reference databases. Consequently, this limitation may affect the generalizability of the study’s recommendations, as they may not be applicable in resource-limited settings. Computational resources and specific scientific issues should also be considered when selecting the optimal combination of reference database and CS in real-world studies.

## Methods

### Generation of simulated metagenomic sequences

Simulated metagenomic datasets were generated using the “iss generate” function in InsilicoSeq (1.6.0.0; Gourlé et al. 2019). Briefly, a metagenomic dataset consisting of 10 million paired-end reads per sample that follow log-normal abundance distribution was generated using the HiSeq model. A composite sample was created by combining 20 random bacterial genomes from NCBI RefSeq so that the relative abundance of each bacterium present in the sample could be calculated. To eliminate random error, a set of 10 samples was created and subsequently used for taxonomic classification.

### Construction of reference database

#### Minikraken v1

A pre-built 8 GB (index size) database was created through a process of subsampling from complete bacteria, archaea and viral genomes in the RefSeq database (March 2020). The Minikraken v1 database offers a streamlined alternative for environments with limited computational resources or for preliminary analyses that require fast, less resource-intensive processing. Despite its small size, it represents a broad microbial diversity and provides accurate taxonomic classification for a wide range of organisms.

#### Standard-16

The Standard-16 database (16 GB, index size) contains archaeal, bacterial, viral, plasmid, humans and UniVec_Core in the RefSeq database (Oct. 9, 2023). The size of the generated database was limited with the option “-max-db-size”.

#### Standard

The standard database (70 GB) is the default database used in Kraken2, which is created based on taxonomic information and complete genomes in the RefSeq database for the bacterial, archaeal and viral domains as well as the human genome and a collection of known vectors (UniVec_Core). It requires ~ 70 GB of RAM to perform a search and ~ 160 GB of disk space to create.

#### nt

The nt database was created in May 2023. It includes GenBank, RefSeq, TPA and PDB. It is very large (480 GB, index size) and is intended to contain a wide range of nucleotide sequences and provide comprehensive coverage for taxonomic classification. The size of the nt database requires considerable computational resources for effective querying and analysis.

#### GTDB r202

The Genome Taxonomy Database (GTDB; 230 GB) is an online resource developed for the systematic classification of bacteria and archaea based on genome phylogeny. GTDB r202 is a special version containing 254,090 bacterial and 4,316 archaeal genomes (April 27, 2021).

### Taxonomic classification using Kraken2 and Bracken

The reference databases were created with ‘kraken2-build –download-taxonomy –db bacteria’, ‘kraken2-build –download-library NAME –db bacteria’ and ‘kraken2-build -build –db NAME’, where NAME specified different reference databases. Then the simulated metagenomic datasets were classified with ‘kraken2 –use-names –threads 20 –db NAME –confidence CS –report REPORT –paired SAMPLE_R1.fastq.gz SAMPLE_R2.fastq.gz > SAMPLE_.kraken’, where CS was 0, 0.2, 0.4, 0.6, 0.8 or 1.0, REPORT denoted a Kraken2 report file and SAMPLE indicated a simulated dataset. The classification rate and relative abundance of each taxon were also calculated using Bracken (Lu et al. 2017), first with ‘bracken-build -d NAME’ and then with ‘bracken -d NAME -i REPORT -o PHYLUM.txt -l P’ and ‘bracken -d NAME -i REPORT -o SPECIES.txt -l S’, where P indicates the phylum and S indicates the species.

### Calculation and statistical analysis

Precision, recall, and F1 score are key metrics used to evaluate the performance of classification in Kraken2 calculated as follows:

$${\text{Precision = }}\frac{{{\text{True}}\,{\text{positive}}}}{{{\text{True}}\,{\text{positive + False}}\,{\text{positive}}}}$$

$${\text{Recall}} = \frac{{{\text{True}}\;{\text{positive}}}}{{{\text{True}}\;{\text{positive + False}}\;{\text{negative}}}}$$

$$F1\;{\text{score}} = 2 \times \frac{{{\text{Precision}} \times {\text{Recall}}}}{{{\text{Precision}} + {\text{Recall}}}}$$

In addition to the above parameters, the difference between the calculated relative abundance at different CS and the true relative abundance was also assessed. The results were analyzed using Kruskal–Wallis in R Studio (version 4.2.1), applying Dunn’s test to perform multiple comparisons. *P*-values were adjusted using the Benjamini–Hochberg method (Benjamini and Hochberg 1995). A significant difference was found at *P* < 0.05.

## Supplementary Information

Below is the link to the electronic supplementary material.

Supplementary file1 (DOCX 28 KB)

## Data Availability

The simulated metagenomic datasets have been deposited in the NCBI Sequence Read Archive (SRA) database under the accession number of PRJNA1061831.

## Code availability

All codes produced by this manuscript have been deposited in the GitHub repository (https://github.com/LIUYUNLONG01/Kraken2).
