# Supplementary material for: Impact of database choice and confidence score on the performance of taxonomic classification using Kraken2
Source: aBIOTECH. 2024 Jul 31;5(4):465–75. doi: 10.1007/s42994-024-00178-0 (PMC11624175; doi:10.1007/s42994-024-00178-0)
Supplement: Supplementary file 1 — Supplementary file1 (DOCX 28 KB) [file 42994_2024_178_MOESM1_ESM.docx]

**Supplementary Information**

| Table 1. Impact of reference database and confidence score on classification rate | | | | | | | | |
| --- | --- | --- | --- | --- | --- | --- | --- | --- |
| Databases | CS 0 | CS 0.2 | CS 0.4 | CS 0.6 | CS 0.8 | CS 1.0 | SEM | *P*-values |
| Minikraken | 0.80^aB^ | 0.54^abB^ | 0.01^bB^ | 0^cB^ | 0^cC^ | 0^cC^ | 0.043 | <0.001 |
| Standard-16 | 0.98^aA^ | 0.54^aB^ | 0^bB^ | 0^bB^ | 0^bC^ | 0^bC^ | 0.050 | <0.001 |
| Standard | 0.92^aA^ | 0.88^aA^ | 0.86^aA^ | 0.83^abA^ | 0.73^bAB^ | 0.46^cAB^ | 0.023 | <0.001 |
| nt | 0.97^aA^ | 0.94^aA^ | 0.93^aA^ | 0.91^abA^ | 0.81^bA^ | 0.52^cA^ | 0.022 | <0.001 |
| GTDB r202 | 0.92^aA^ | 0.86^aA^ | 0.81^abA^ | 0.72^bcA^ | 0.54^cdB^ | 0.24^dB^ | 0.032 | <0.001 |
| SEM | 0.012 | 0.042 | 0.057 | 0.054 | 0.047 | 0.029 |  |  |
| *P*-values | <0.001 | <0.001 | <0.001 | <0.001 | <0.001 | <0.001 |  |  |

^abcd^ Data in the same row with different superscripts indicate a significant difference with various confidence scores according to Dunn’s test (*P* < 0.05).

^ABC^ Data in the same column with different superscripts indicate a significant difference with various reference databases according to Dunn’s test (*P* < 0.05).

| Table 2. Impact of databases choice and confidence score on classification precision | | | | | | | | |
| --- | --- | --- | --- | --- | --- | --- | --- | --- |
| Databases | CS 0 | CS 0.2 | CS 0.4 | CS 0.6 | CS 0.8 | CS 1.0 | SEM | *P*-values |
| Phylum | | | | | | | | |
| Minikraken | 0.45^bAB^ | 0.92^aA^ | 0.94^aA^ | 0^cB^ | 0^cB^ | 0^cB^ | 0.057 | <0.001 |
| Standard-16 | 0.71^aA^ | 0.90^aA^ | 0.93^aA^ | 0^bB^ | 0^bB^ | 0^bB^ | 0.060 | <0.001 |
| Standard | 0.36^bB^ | 0.87^aA^ | 0.91^aA^ | 0.91^aA^ | 0.93^aA^ | 0.93^aA^ | 0.040 | <0.001 |
| nt | 0.16^bC^ | 0.76^aAB^ | 0.86^aA^ | 0.87^aA^ | 0.87^aA^ | 0.87^aA^ | 0.043 | <0.001 |
| GTDB r202 | 0.11^dC^ | 0.49^cB^ | 0.60^cB^ | 0.69^bcA^ | 0.78^abA^ | 0.93^aA^ | 0.038 | <0.001 |
| SEM | 0.039 | 0.038 | 0.034 | 0.063 | 0.064 | 0.067 |  |  |
| *P*-values | <0.001 | <0.001 | <0.001 | <0.001 | <0.001 | <0.001 |  |  |
| Species | | | | | | | | |
| Minikraken | 0.05^bB^ | 0.56^abAB^ | 0.84^aA^ | 0^cC^ | 0^cC^ | 0^cB^ | 0.047 | <0.0001 |
| Standard-16 | 0.10^aA^ | 0.84^aA^ | 0.88^aA^ | 0^bC^ | 0^bC^ | 0^bB^ | 0.056 | <0.0001 |
| Standard | 0.04^cB^ | 0.33^bC^ | 0.48^bBC^ | 0.59^aAB^ | 0.70^aAB^ | 0.79^aA^ | 0.043 | <0.0001 |
| nt | 0.04^cB^ | 0.46^bcBC^ | 0.67^abAB^ | 0.76^aA^ | 0.84^aA^ | 0.87^aA^ | 0.048 | <0.0001 |
| GTDB r202 | 0.01^deC^ | 0.07^cdD^ | 0.13^cC^ | 0.20^bcB^ | 0.33^abB^ | 0.69^aA^ | 0.033 | <0.0001 |
| SEM | 0.006 | 0.046 | 0.051 | 0.050 | 0.056 | 0.062 |  |  |
| *P*-values | <0.001 | <0.001 | <0.001 | <0.001 | <0.001 | <0.001 |  |  |

^abc^ Data in the same row with different superscripts indicate a significant difference with various confidence scores according to Dunn’s test (*P* < 0.05).

^ABC^ Data in the same column with different superscripts indicate a significant difference with various reference databases according to Dunn’s test (*P* < 0.05).

| Table 3. Impact of databases choice and confidence score on classification recall | | | | | | | | |
| --- | --- | --- | --- | --- | --- | --- | --- | --- |
| Databases | CS 0 | CS 0.2 | CS 0.4 | CS 0.6 | CS 0.8 | CS 1.0 | SEM | *P*-values |
| Phylum | | | | | | | | |
| Minikraken | 0.92^a^ | 0.85^a^ | 0.83^a^ | 0^bB^ | 0^bB^ | 0^bB^ | 0.059 | <0.001 |
| Standard-16 | 0.96^a^ | 0.93^a^ | 0.93^a^ | 0^bB^ | 0^bB^ | 0^bB^ | 0.062 | <0.001 |
| Standard | 0.88 | 0.84 | 0.84 | 0.84^A^ | 0.84^A^ | 0.81^A^ | 0.021 | 0.994 |
| nt | 0.86 | 0.84 | 0.84 | 0.84^A^ | 0.84^A^ | 0.82^A^ | 0.024 | 0.999 |
| GTDB r202 | 1.00 | 1.00 | 1.00 | 1.00^A^ | 0.98^A^ | 0.98^A^ | 0.004 | 0.540 |
| SEM | 0.018 | 0.024 | 0.025 | 0.065 | 0.065 | 0.064 |  |  |
| *P*-values | 0.078 | 0.052 | 0.050 | <0.001 | <0.001 | <0.001 |  |  |
| Species | | | | | | | | |
| Minikraken | 0.73^aB^ | 0.72^aB^ | 0.54^aB^ | 0^bB^ | 0^bB^ | 0^bB^ | 0.047 | <0.001 |
| Standard-16 | 0.89^aA^ | 0.86^aA^ | 0.45^aB^ | 0^bB^ | 0^bB^ | 0^bB^ | 0.055 | <0.001 |
| Standard | 0.83^AB^ | 0.82^AB^ | 0.80^A^ | 0.80^A^ | 0.79^A^ | 0.79^A^ | 0.043 | 0.894 |
| nt | 0.89^A^ | 0.87^A^ | 0.86^A^ | 0.86^A^ | 0.85^A^ | 0.80^A^ | 0.030 | 0.166 |
| GTDB r202 | 0.81^AB^ | 0.81^AB^ | 0.81^A^ | 0.81^A^ | 0.81^A^ | 0.81^A^ | 0.027 | 0.999 |
| SEM | 0.031 | 0.033 | 0.039 | 0.063 | 0.062 | 0.061 |  |  |
| *P*-values | 0.003 | 0.035 | 0.001 | <0.001 | <0.001 | <0.001 |  |  |

^ab^ Data in the same row with different superscripts indicate a significant difference with various confidence scores according to Dunn’s test (*P* < 0.05).

^ABC^ Data in the same column with different superscripts indicate a significant difference with various reference databases according to Dunn’s test (*P* < 0.05).

| Table 4. Impact of databases choice and confidence score on classification F1 score | | | | | | | | |
| --- | --- | --- | --- | --- | --- | --- | --- | --- |
| Databases | CS 0 | CS 0.2 | CS 0.4 | CS 0.6 | CS 0.8 | CS 1.0 | SEM | *P*-values |
| Phylum | | | | | | | | |
| Minikraken | 0.59^aAB^ | 0.88^aA^ | 0.87^aAB^ | 0^bB^ | 0^bB^ | 0^bB^ | 0.055 | <0.001 |
| Standard-16 | 0.79^aA^ | 0.91^aA^ | 0.93^aA^ | 0^bB^ | 0^bB^ | 0^bB^ | 0.060 | <0.001 |
| Standard | 0.48^bB^ | 0.84^aA^ | 0.87^aAB^ | 0.87^aA^ | 0.87^aA^ | 0.85^aA^ | 0.031 | 0.018 |
| nt | 0.26^bC^ | 0.79^aA^ | 0.84^aAB^ | 0.84^aA^ | 0.84^aA^ | 0.83^aA^ | 0.038 | <0.001 |
| GTDB r202 | 0.20^dC^ | 0.65^cB^ | 0.74^bcB^ | 0.81^bcA^ | 0.86^abA^ | 0.94^aA^ | 0.034 | <0.001 |
| SEM | 0.038 | 0.030 | 0.027 | 0.062 | 0.063 | 0.065 |  |  |
| *P*-values | <0.001 | 0.006 | 0.026 | <0.001 | <0.001 | <0.001 |  |  |
| Species | | | | | | | | |
| Minikraken | 0.08^bB^ | 0.62^aAB^ | 0.65^aA^ | 0^cC^ | 0^cC^ | 0^cB^ | 0.041 | <0.001 |
| Standard-16 | 0.18^bA^ | 0.85^aA^ | 0.58^abA^ | 0^cC^ | 0^cC^ | 0^cB^ | 0.047 | <0.001 |
| Standard | 0.07^dB^ | 0.46^bcB^ | 0.58^bcA^ | 0.67^abcAB^ | 0.74^abA^ | 0.78^aA^ | 0.041 | <0.001 |
| nt | 0.07^bcB^ | 0.58^bB^ | 0.74^abA^ | 0.80^aA^ | 0.84^aA^ | 0.83^aA^ | 0.046 | <0.001 |
| GTDB r202 | 0.02^eC^ | 0.12^deC^ | 0.22^cdB^ | 0.32^bcB^ | 0.46^abB^ | 0.73^aA^ | 0.034 | <0.001 |
| SEM | 0.011 | 0.044 | 0.039 | 0.053 | 0.056 | 0.061 |  |  |
| *P*-values | <0.001 | <0.001 | <0.001 | <0.001 | <0.001 | <0.001 |  |  |

^abcde^ Data in the same row with different superscripts indicate a significant difference with various confidence scores according to Dunn’s test (*P* < 0.05).

^ABC^ Data in the same column with different superscripts indicate a significant difference with various reference databases according to Dunn’s test (*P* < 0.05).

| Table 5. Impact of databases choice and confidence score on difference of bacterial abundance | | | | | | | | |
| --- | --- | --- | --- | --- | --- | --- | --- | --- |
| Databases | CS 0 | CS 0.2 | CS 0.4 | CS 0.6 | CS 0.8 | CS 1.0 | SEM | *P*-values |
| Phylum | | | | | | | | |
| Minikraken | 5.08^bA^ | 5.73^bA^ | 5.96^bA^ | 22.22^aA^ | 22.22^aA^ | 22.22^aA^ | 1.113 | <0.001 |
| Standard-16 | 1.86^cD^ | 2.15^bCD^ | 2.74^bC^ | 22.22^aA^ | 22.22^aA^ | 22.22^aA^ | 1.157 | <0.001 |
| Standard | 3.03^B^ | 3.85^B^ | 4.39^B^ | 4.74^B^ | 4.91^B^ | 4.90^C^ | 0.317 | 0.057 |
| nt | 2.16^dC^ | 2.83^cdC^ | 3.94^bcBC^ | 5.51^bB^ | 6.91^abB^ | 9.84^aB^ | 0.445 | <0.001 |
| GTDB | 2.95^dBC^ | 3.37^cdBC^ | 3.58^bcdC^ | 3.83^abcB^ | 4.22^abB^ | 6.74^aBC^ | 0.292 | <0.001 |
| SEM | 0.320 | 0.336 | 0.323 | 1.143 | 1.142 | 1.138 |  |  |
| *P*-values | <0.001 | <0.001 | 0.023 | <0.001 | <0.001 | <0.001 |  |  |
| Species | | | | | | | | |
| Minikraken | 2.64^cA^ | 3.17^cA^ | 4.58^bA^ | 5.44^aA^ | 5.44^aA^ | 5.44^aA^ | 0.166 | <0.001 |
| Standard-16 | 1.21^cD^ | 1.84^bCD^ | 6.04^aA^ | 5.44^aA^ | 5.44^aA^ | 5.44^aA^ | 0.174 | <0.001 |
| Standard | 1.73^dB^ | 1.99^cdC^ | 1.91^cdC^ | 2.12^bcC^ | 2.52^abC^ | 3.21^aC^ | 0.121 | <0.001 |
| nt | 1.26^eC^ | 1.63^dD^ | 1.92^cdC^ | 2.19^cC^ | 2.68^bC^ | 4.14^aB^ | 0.127 | <0.001 |
| GTDB r202 | 1.76^eB^ | 2.42^dB^ | 3.17^cB^ | 4.03^bB^ | 5.01^aB^ | 6.37^aA^ | 0.174 | <0.001 |
| SEM | 0.118 | 0.126 | 0.183 | 0.173 | 0.179 | 0.209 |  |  |
| *P*-values | <0.001 | <0.001 | <0.001 | <0.001 | <0.001 | <0.001 |  |  |

^abcd^ Data in the same row with different superscripts indicate a significant difference with various confidence scores according to Dunn’s test (*P* < 0.05).

^ABCD^ Data in the same column with different superscripts indicate a significant difference with various reference databases according to Dunn’s test (*P* < 0.05).
